# Association of Genetic Polymorphisms and Serum Levels of miR-1-3p with Postoperative Mortality following Abdominal Aortic Aneurysm Repair

**DOI:** 10.3390/jcm12030946

**Published:** 2023-01-26

**Authors:** Tan Li, Bo Jiang, Yijun Wu, Jun Yang, Chunyan Ma, Yuan Yuan

**Affiliations:** 1 Department of Cardiovascular Ultrasound, The First Hospital of China Medical University, Shenyang 110001, China; 2 Clinical Medical Research Center of Imaging in Liaoning Province, The First Hospital of China Medical University, Shenyang 110001, China; 3 Department of Vascular Surgery, The First Hospital of China Medical University, Shenyang 110001, China; 4 Tumor Etiology and Screening Department of Cancer Institute and General Surgery, The First Hospital of China Medical University, Shenyang 110001, China; 5 Key Laboratory of Cancer Etiology and Prevention in Liaoning Education Department, The First Hospital of China Medical University, Shenyang 110001, China

**Keywords:** abdominal aortic aneurysm, miRNA, polymorphism, prognosis, mortality

## Abstract

Background: Several miRNAs have been implicated in the clinical outcomes of cardiovascular disorders, but the role of miR-1-3p in abdominal aortic aneurysm (AAA) prognosis remains unclear. This study aimed to investigate the correlation of single nucleotide polymorphisms (SNPs) in pri-miR-1-3p and mature miR-1-3p expression with postoperative mortality of AAA patients. Methods: A total of 230 AAA patients who received AAA repair were recruited and followed up for 5 years. SNP genotyping was carried out using KASP method and relative expression of serum miR-1-3p was measured with qRT-PCR. Results: Multivariate Cox regression analyses showed that both rs2155975 and rs4591246 variant genotypes were associated with increased all-cause mortality of postoperative AAA patients after adjusting possible confounders. Patients who died tended to have lower baseline miR-1-3p expression (overall and for age < 65 years, aneurysm-related death or cardiac death subgroup) when compared to alive patients; further Cox regression yielded an independent relationship of preoperative low serum miR-1-3p levels with incidents of all-cause death. Patients carrying rs2155975 AG + GG or rs4591246 AG + AA genotype had a higher ratio of low miR-1-3p levels in contrast to those with AA or GG genotype, respectively. The Kaplan–Meier survival curves suggested that the combined genotype in rs2155975 or rs4591246 and low miR-1-3p levels could decrease the overall survival of AAA patients during 5-year follow-up. Conclusions: This pilot study demonstrated the importance of rs2155975 and rs4591246 polymorphisms and baseline serum miR-1-3p levels as promising markers to predict mortality among patients following AAA repair.

## 1. Introduction

Abdominal aortic aneurysm (AAA) is a potentially life-threatening cardiovascular disease typically described as a focal weakening and dilatation of the abdominal aorta [1]. The major epidemiological risk factors for AAA include male sex, older age, smoking, hypertension and dyslipidemia [1,2]. Aortic wall inflammation, vascular smooth muscle cell (VSMC) apoptosis and extracellular matrix degradation are the key pathological hallmarks in the progress of AAA formation [3,4]. At present, a patient with large AAA or symptomatic or ruptured AAA of any size should be referred for AAA repair by either open aneurysm repair (OAR) or endovascular aneurysm repair (EVAR) [5,6]. Despite an early benefit being realized with EVAR, no difference was observed in all-cause mortality between the two procedures in the mid- or longer-term follow-up for AAA patients [7,8,9]. Notably, a genetic component plays a crucial role in predicting the risk of AAA occurrence and adverse outcomes after therapy [10]. Although clinical detection methods and treatment strategies have been improved, elderly patients still suffer from major postoperative complications and poor quality of life, and the 5-year survival rate for AAA patients remains low [11,12]. There is an urgent need to identify preoperative determinants of future mortality in AAA patients, which may optimize risk stratification and improve clinical decision-making and management.

As a major type of genetic variant, a series of single nucleotide polymorphisms (SNPs) have been considered candidate biomarkers of genetic background to predict risk, progression and prognosis of diseases [13]. MicroRNAs (miRNAs) are small non-protein coding single-stranded RNAs that are derived from long primary miRNAs (pri-miRNAs) and exert the main function of post-transcriptional gene regulation [14,15]. Variants in non-coding regions account for more than one-third of the variants identified by genome-wide association studies [16]. The presence of SNP in miRNA genes may have an influence on its transcription, maturation and miRNA-mRNA interaction, and explain the aberrant miRNA expression in multiple diseases [17,18]. It has become evident that functional miRNA polymorphisms may confer genetic susceptibility to certain diseases and be related to the clinical outcomes of patients with these disorders [19,20,21]. However, reports assigning SNPs in miRNA to human AAA prognosis are still lacking.

Recently, miR-1-3p has been shown to be a promising and effective biomarker implicated in the pathogenesis of cardiovascular events [22,23]. MiR-1-3p was downregulated and correlated with cardiac function in human hypertrophic cardiomyopathy [24]. In VSMCs, miR-1 has been reported to be an important modulator of gene expression and cell contractility, and acts a critical role in regulating pulmonary vascular remodeling of pulmonary arterial hypertension patients [25,26]. Although accumulating evidence suggests that changes in the expression of several circulating miRNAs are linked with the adverse outcomes of AAA patients [27,28], the effect of serum miR-1-3p expression on the prognosis of patients receiving AAA surgery has not been reported.

In this prospective study, we sought to further evaluate whether the pri-miR-1-3p polymorphisms along with serum miR-1-3p expression predicted all-cause mortality in patients undergoing AAA repair, based on a Chinese population. These data may aid in the timing of clinical surgical intervention, counseling of patients on expected outcomes, and improving late survival.

## 2. Materials and Methods

### 2.1. Study Population

We enrolled a total of 230 AAA patients who underwent a successful EVAR or OAR procedure in the First Hospital of China Medical University and were successfully followed up for 5 years. Preoperative computed tomography angiography was applied to diagnose the patients and assess the maximum AAA diameter. Exclusion criteria included the subjects with traumatic AAA, previous aortic surgery, congenital disorder, malignant tumor, infectious disease, hematological disease, severe hepatic or renal dysfunction (end-stage renal disease requiring dialysis treatment) or severe heart disease (congestive heart failure, hypertrophic obstructive cardiomyopathy, severe valvular stenosis or regurgitation and/or life-threatening arrhythmias). Patients who had no follow-up information or died within 30 days after surgery were also excluded. Blood clots and serum samples were collected from AAA patients before surgery. Baseline demographic data, comorbidities and surgical information were obtained from the medical records. The study was approved by the Ethics Committee of the First Hospital of China Medical University (Shenyang, China). Informed consent was obtained from each participant.

The median follow-up time was 5 years and the primary outcome of interest was all-cause mortality within 5 years after AAA surgery. The overall survival was measured as the period from the time of surgical treatment to the date of death from any cause or the last follow-up. Patients were followed up for a maximum of 5 years via annual telephone contact or clinical visits, and the most recent follow-up assessments were completed on 17 February 2022.

### 2.2. SNP Selection

We utilized the NCBI dbSNP database and Haploview software 4.2 to select tagger SNPs in pri-miR-1-3p. The selection criteria were: (1) minor allele frequency (MAF) > 0.05 in the Chinese Han population (CHB); (2) lack of linkage disequilibrium (*r^2^* < 0.8) between tagger SNPs; and (3) following the Hardy–Weinberg equilibrium (HWE, *p* > 0.05). Consequently, three tagger SNPs were selected as research targets, including rs2155975, rs4591246 and rs9989532. Based upon the SNPinfo website, it is predicted that these tagger SNPs may affect the activity of transcription factor binding sites.

### 2.3. DNA Extraction and Genotyping

Genomic DNA was extracted from blood clots using a standard phenol-chloroform method. The concentration and quality of DNA were checked with NanoDrop 2000 (Thermo Fisher Scientific, Waltham, USA). SNP genotyping was performed by Baygene Biotechnology Company Limited (Shanghai, China) with the KASP method using SNPLine platform (LGC, United Kingdom). Additionally, 10% of the DNA samples were randomly selected for duplicate genotyping, and the results showed 100% concordance.

### 2.4. Quantitative Real-Time PCR (qRT-PCR)

Total RNA was isolated from serum with Trizol reagent (Thermo Fisher Scientific, Waltham, USA) according to the manufacturer’s protocol. We used the miRcute miRNA First-Strand cDNA Synthesis Kit (Tiangen, Beijing, China) and miRcute miRNA SYBR Green qPCR Detection Kit (Tiangen, Beijing, China) for reverse transcription and quantitative detection, respectively. The primer sequences for qRT-PCR were as follows: miR-1-3p, forward 5′-TGGAATGTAAAGAAGTATGTAT-3′ and reverse 5′-CGCTTCACGAATTTGCGTG-3′; U6, forward 5′-CTCGCTTCGGCAGCACA-3′ and reverse 5′-AACGCTTCACGAATTTGCGT-3′. The qRT-PCR was performed under the following conditions: 95 °C for 15 min, followed by 40 cycles of 94 °C for 20 s and 60 °C for 34 s. U6 was adopted as the internal control and the relative expression of miR-1-3p was determined with the 2^−ΔΔCT^ method.

### 2.5. Statistical Analysis

All statistical analyses were performed with SPSS 23.0 software. Continuous data were described as mean ± standard deviation (SD), and categorical data were presented as counts and percentages. Normally distributed data were analyzed by Student’s *t*-test, nonparametric data were compared by Mann–Whitney U-test and categorical data were tested with Chi-square test. A receiver operating characteristic (ROC) curve was employed to determine a cut-off value of serum miR-1-3p levels associated with 5-year mortality. The overall survival was analyzed with the Kaplan–Meier method and differences were compared with log-rank test. The effects of SNPs and miR-1-3p expression on all-cause mortality were evaluated using univariate and multivariate Cox regression models by calculating hazard ratios (HRs) and their 95% confidence intervals (95% CIs). A two-tailed *p* value < 0.05 was deemed statistically significant. Additionally, the dominant and recessive models were separately defined as heterozygote + homozygote variant vs. homozygote wild and homozygote variant vs. heterozygote + homozygote wild.

## 3. Results

### 3.1. Baseline Characteristics of the Study Population

Detailed demographic and clinical characteristics of the total enrolled population are listed in Table 1. Overall, 57 patients (24.8%) died during the follow-up period of 5 years consisting of 11 aneurysm-related deaths and 9 cardiac death events. Patients who died were older or had larger AAA diameters or a higher ratio of renal insufficiency compared with those who survived (all *p* < 0.05). Then, the prognostic impact of clinical features on mortality was evaluated by univariate Cox regression analysis and only variables that achieved *p* < 0.10 were included in the subsequent multivariate Cox regression models (Table S1).

### 3.2. Association of the Studied SNPs with AAA Prognosis

**Figure 1:**
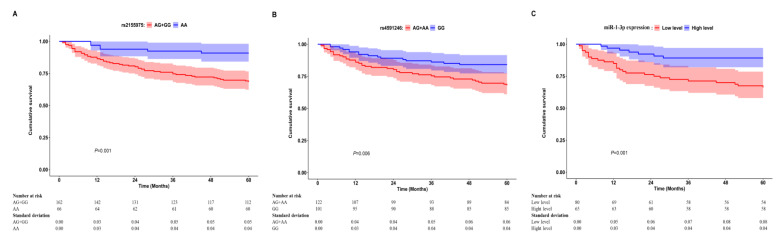
Kaplan–Meier estimates of the cumulative survival in patients after AAA repair according to SNP rs2155975 (**A**) and rs4591246 (**B**) as well as miR-1-3p expression (**C**). Shaded region indicates 95% confidence interval (CI).

Based on all enrolled AAA patients, the relationship of rs2155975, rs4591246 and rs9989532 polymorphisms with postoperative all-cause mortality was estimated in univariate and multivariate analyses. Compared to homozygous wild-type genotype, the heterozygous genotype, homozygous variant genotype and dominant model of either rs2155975 or rs4591246 could significantly increase the risk of all-cause death in multivariate Cox analysis with adjustment for age, renal insufficiency, maximum AAA diameter, presence of surgical complications and presence of postoperative renal dysfunction (for rs2155975, AG vs. AA: *p* = 0.004, HR = 3.591, 95% CI = 1.489-8.658, GG vs. AA: *p* = 0.010, HR = 3.590, 95% CI = 1.358-9.487, dominant model: *p* = 0.003, HR = 3.668, 95% CI = 1.560-8.627; for rs4591246, AG vs. GG: *p* = 0.016, HR = 2.117, 95% CI = 1.150-3.898, AA vs. GG: *p* = 0.039, HR = 2.737, 95% CI = 1.043-5.400, dominant model: *p* = 0.007, HR = 2.225, 95% CI = 1.240-3.992) (Table 2). Moreover, Cox regression models showed no statistical association between rs9989532 polymorphism and AAA prognosis. The Kaplan–Meier survival curves indicated that the dominant model in rs2155975 and rs4591246 could significantly decrease the overall survival of AAA patients in contrast to homozygous wild-type genotype (all *p* < 0.05) (Figure 1A,B).

### 3.3. Association of Serum miR-1-3p Expression with AAA Prognosis

A total of 145 serum samples with SNP genotyping information were available for miR-1-3p detection. In the overall comparison, patients who died due to all causes were more likely to have lower preoperative miR-1-3p levels than those who survived (0.89 ± 0.60 vs. 1.27 ± 0.92, *p* = 0.011) (Table 3). The stratified analyses showed that baseline miR-1-3p expression was significantly lower in dead patients when compared with alive subjects in age < 65 years, aneurysm-related death and cardiac death subgroups (all *p* < 0.05) (Table 3). Meanwhile, Cox regression analyses suggested that increased miR-1-3p expression was correlated with a reduced risk of all-cause death even after adjusting for age, renal insufficiency, maximum AAA diameter, presence of surgical complications and presence of postoperative renal dysfunction (*p* = 0.030, HR = 0.496 per unit increase, 95% CI = 0.264–0.933). In addition, an ideal cut-off value of miR-1-3p levels determined by the ROC curve against mortality was 1.03 with the area under the curve of 0.643 (*p* = 0.012, 95% CI = 0.541–0.746). Using this cut-off value, we differentiated patients into low level (<1.03) group and high level (≥1.03) group. With respect to this classification, preoperative low levels of serum miR-1-3p were found to be significantly associated with increased all-cause mortality of AAA patients in both univariate and multivariate Cox models (all *p* < 0.05) (Table 4). The corresponding survival curve revealed that patients with low miR-1-3p levels had a worse overall survival when compared to those with high miR-1-3p levels (*p* = 0.001) (Figure 1C).

### 3.4. Effects of rs2155975 and rs4591246 on the Expression of Serum miR-1-3p

To explore the influence of two significant polymorphisms on gene expression, we assessed the serum levels of miR-1-3p in patients carrying different genotypes of rs2155975 or rs4591246. Patients with AG + GG genotype of rs2155975 exhibited a much higher proportion of low miR-1-3p levels than those with AA genotype (*p* = 0.029) (Table 5). Similarly, in the case of rs4591246, carriers with AG + AA genotype displayed an elevated ratio of low miR-1-3p levels in comparison to those with GG genotype (*p* = 0.029) (Table 5). However, when miR-1-3p expression was taken as a continuous variable, rs2155975 and rs4591246 had no statistical impact on serum miR-1-3p levels in AAA patients (Table 5).

## 4. Discussion

With the support of molecular biomarkers, precision medicine has advanced prognostic prediction. Here, we present the results of a 5-year prospective study that elucidates the influence of SNPs in pri-miR-1-3p as well as serum miR-1-3p expression on AAA prognosis, with a specific focus on all-cause mortality under surgical therapy. Our findings provide a novel clinical tool to assess the risk of subsequent death events after AAA repair, which may help guide the management of AAA patients.

The long-term mortality results for AAA patients after surgery with follow-up ranging from 3 to 15 years have been reported, and the data taken together exhibit a trend of approximately 5% all-cause mortality with each year of postoperative follow-up [29,30]. Our study presented an overall mortality rate of 24.8% at a maximum follow-up of 5 years, which was in line with the above trend. It has been demonstrated that SNPs located in pri-miRNAs may interfere with the processing of miRNAs and affect mature miRNA expression as a consequence, which leads to altered biological functions and thereby influences the survival outcome of patients [31,32,33]. The predictive utility of pri-miRNA SNPs for overall survival has been studied in several types of human disease [20,21,33]. In our study, Cox regression models revealed that heterozygous genotype and dominant model of both rs2155975 and rs4591246 could increase 5-year all-cause mortality of AAA patients after surgical intervention even controlling for possible confounding factors. Further survival analysis demonstrated that the combined variant genotype of either rs2155975 or rs4591246 was closely related to worse overall survival in comparison with homozygous wild-type genotype. To some extent, these data might provide a clue for analyzing the prognostic value of rs2155975 and rs4591246 in predicting the mortality after AAA repair. However, the mechanisms underlying how they are involved in postoperative AAA mortality are complicated and need more in-depth research to reveal them.

In fact, a number of miRNAs have been identified as serum biomarkers with high potential to be used in clinical diagnosis in a stable form, or provide accurate prognosis for survival in patients with cardiovascular disorders [14,34]. Inflammation plays a central role in AAA pathogenesis, and a growing body of studies has noted that AAA patients with elevated levels of systemic inflammatory markers before surgery tended to have more frequent postoperative complications and major adverse events, including all-cause death [35,36,37]. As a muscle-specific miRNA, miR-1-3p has been discovered to participate in modulating inflammatory responses in a variety of biological processes and pathologies [38,39,40]. Badacz et al. revealed that circulating miR-1-3p appeared as a prognostic factor of secondary cardiovascular events during follow-up [23]. Navickas et al. suggested that miR-1 could regulate the endothelial function and angiogenesis, apoptosis as well as cardiac myocyte differentiation, and its circulating abundance was markedly correlated with worse myocardial infarction outcome [34]. In the current study, it was interesting to note that AAA patients who died due to all-cause, aneurysm-related or cardiac events at the follow-up exhibited lower preoperative levels of serum miR-1-3p compared with subjects who survived. In addition, we observed that reduced miR-1-3p expression was a strong predictor of 5-year all-cause mortality risk of patients after AAA repair, independent of potential confounders. The survival curve indicated that AAA patients with low levels of serum miR-1-3p versus high levels were more likely to have shorter overall survival. Therefore, determining serum miR-1-3p at baseline could be useful in implementing measures to improve their clinical outcomes.

Furthermore, we determined that the presence of genetic variation in pri-miR-1-3p had a remarkable effect on the production of mature miR-1-3p. In detail, AAA patients with rs2155975 AG + GG or rs4591246 AG + AA genotype displayed a much higher proportion of low miR-1-3p levels in serum than carriers of the homozygous wild-type genotype. According to SNPinfo database, rs2155975 and rs4591246 were putatively located in the transcription factor binding sites of pri-miR-1-3p, where they might regulate the binding activity and impact the expression of mature miR-1-3p. Taken together, it is reasonable to suppose that rs2155975 and rs4591246 may be functional SNPs and contribute to the long-term mortality of AAA patients after surgery via modifying miR-1-3p expression levels. In the same way, some miRNA SNPs have been recognized to affect disease prognosis through regulating the expression of mature miRNAs [21,41,42].

Some limitations should be acknowledged. First, we included tagger SNPs located in pri-miR-1-3p and more SNPs in multiple miRNAs needed to be detected. Second, despite of our efforts to collect necessary data, certain variables that might affect patients’ survival after surgery were unavailable. Third, this research was based upon a single institution and the number of samples was relatively small, which limited the evaluation of cause-specific mortality such as aneurysm-related death and cardiac death. Therefore, our results should be confirmed in a multicenter study with a larger sample size and longer follow-up.

## 5. Conclusions

In summary, this study discovered that miR-1-3p polymorphisms and low expression were closely linked to increased 5-year all-cause mortality and shorter survival of patients undergoing AAA repair. The combined variant genotype of either rs2155975 or rs4591246 could affect the expression of serum miR-1-3p. Thus, SNP rs2155975 and rs4591246 and miR-1-3p expression might be used as prognostic indicators to predict postoperative death events among AAA patients, which could be helpful in the preoperative counselling and decision-making process.

## Figures and Tables

**Table 1:** Baseline characteristics of the total enrolled population.

Variable	Alive Group (*n* = 173)	Dead Group (*n* = 57)	*p* Value
Age, years	66.36 ± 9.84	71.75 ± 9.13	**<0.001**
<65 years, n (%)	68 (39.3%)	12 (21.1%)	**0.016**
≥65 years, n (%)	105 (60.7%)	45 (78.9%)	
Male sex, n (%)	137 (79.2%)	48 (84.2%)	0.449
Smoking, n (%)	64 (37.0%)	25 (43.9%)	0.433
Drinking, n (%)	34 (19.7%)	8 (14.0%)	0.431
BMI, kg/m^2^	24.06 ± 3.62	23.40 ± 3.51	0.239
Comorbidities			
Coronary artery disease, n (%)	33 (19.1%)	12 (21.0%)	0.847
Cerebrovascular disease, n (%)	18 (10.4%)	10 (17.5%)	0.165
Peripheral artery disease, n (%)	40 (23.1%)	16 (28.1%)	0.479
Renal insufficiency, n (%)	17 (9.8%)	15 (26.3%)	**0.004**
Atrial fibrillation, n (%)	9 (5.2%)	4 (7.0%)	0.741
Hypertension, n (%)	109 (63.0%)	42 (73.7%)	0.151
Diabetes, n (%)	21 (12.1%)	12 (21.1%)	0.122
Dyslipidemia, n (%)	106 (61.3%)	33 (57.9%)	0.864
Maximum AAA diameter, cm	5.70 ± 1.52	6.41 ± 2.13	**0.010**
<5.0 cm, n (%)	46 (26.6%)	10 (17.5%)	0.213
≥5.0 cm, n (%)	127 (73.4%)	47 (82.5%)	
Presence of surgical complications, n (%)	20 (11.6%)	12 (21.1%)	0.081
Presence of postoperative renal dysfunction, n (%)	8 (4.6%)	6 (10.5%)	0.117
Presence of postoperative respiratory impairment, n (%)	13 (7.5%)	7 (12.3%)	0.283
Presence of reoperation, n (%)	20 (11.6%)	8 (14.0%)	0.643
Surgical type			
EVAR, n (%)	137 (79.2%)	49 (86.0%)	0.333
OAR, n (%)	36 (20.8%)	8 (14.0%)	
Cause of death			
Aneurysm-related death, n (%)	/	11 (19.3%)	/
Cardiac death, n (%)	/	9 (15.8%)	/
Others, n (%)	/	37 (64.9%)	/

Bold values indicate statistically significant *p*-value < 0.05. BMI: body mass index; AAA: abdominal aortic aneurysm; EVAR: endovascular abdominal aortic aneurysm repair; OAR: open aneurysm repair.

**Table 2:** Cox regression analysis for the association between studied SNPs and postoperative mortality of AAA patients through 5-year follow-up.

SNP Genotypes	Alive	Dead	Univariate		Multivariate ^a^	
			*p* Value	HR (95% CI)	*p* Value	HR (95% CI)
rs2155975	*n* = 171	*n* = 57				
AA	60	6		1 (Ref)		1 (Ref)
AG	80	35	**0.003**	3.783 (1.591–8.997)	**0.004**	3.591 (1.489–8.658)
GG	31	16	**0.002**	4.376 (1.711–11.191)	**0.010**	3.590 (1.358–9.487)
AG + GG vs. AA			**0.001**	3.955 (1.697–9.217)	**0.003**	3.668 (1.560–8.627)
GG vs. AG + AA			0.100	1.623 (0.911–2.893)	0.169	1.516 (0.838–2.743)
rs4591246	*n* = 168	*n* = 55				
GG	85	16		1 (Ref)		1 (Ref)
AG	70	30	**0.019**	2.073 (1.130–3.804)	**0.016**	2.117 (1.150–3.898)
AA	13	9	**0.013**	2.806 (1.239–6.355)	**0.039**	2.737 (1.043–5.400)
AG + AA vs. GG			**0.007**	2.213 (1.236–3.960)	**0.007**	2.225 (1.240–3.992)
AA vs. AG + GG			0.089	1.859 (0.910–3.800)	0.107	1.811 (0.880–3.726)
rs9989532	*n* = 172	*n* = 57				
TT	142	47		1 (Ref)		1 (Ref)
TC	27	9	0.938	1.029 (0.504–2.099)	0.615	0.825 (0.390–1.745)
CC	3	1	0.930	1.092 (0.151–7.918)	0.962	1.051 (0.141–7.843)
TC + CC vs. TT			0.923	1.034 (0.523–2.047)	0.646	0.846 (0.416–1.726)
CC vs. TC + TT			0.935	1.086 (0.150–7.848)	0.934	1.088 (0.147–8.064)

Bold values indicate statistically significant *p*-value < 0.05. ^a^, *p* for association was adjusted by age, renal insufficiency, maximum AAA diameter, presence of surgical complications and presence of postoperative renal dysfunction. AAA: abdominal aortic aneurysm; SNP: single nucleotide polymorphism; HR: hazard ratio; CI: confidence interval.

**Table 3:** Baseline serum miR-1-3p levels between alive and dead patients in the overall and stratified comparisons.

	Variable	Alive	Dead	Serum miR-1-3p Expression		
				Alive	Dead	*p* Value
Overall		*n* = 111	*n* = 34	1.27 ± 0.92	0.89 ± 0.60	**0.011**
Age	<65 years	41	6	1.36 ± 1.13	0.44 ± 0.21	**0.008**
	≥65 years	70	28	1.22 ± 0.79	0.98 ± 0.62	0.151
Sex	Male	88	26	1.29 ± 0.96	0.92 ± 0.65	0.065
	Female	23	8	1.21 ± 0.81	0.79 ± 0.42	0.168
AAA size	<5.0 cm	25	6	1.43 ± 0.97	0.86 ± 0.23	0.166
	≥5.0 cm	86	28	1.23 ± 0.91	0.89 ± 0.66	0.075
Surgical type	EVAR	90	26	1.27 ± 0.88	0.98 ± 0.65	0.120
	OAR	21	8	1.28 ± 1.11	0.58 ± 0.28	0.093
Cause of death	Aneurysm-related death	111	9	1.27 ± 0.92	0.57 ± 0.44	**0.026**
	Cardiac death	111	6	1.27 ± 0.92	0.54 ± 0.14	**0.008**
	Others	111	19	1.27 ± 0.92	1.15 ± 0.64	0.566

Bold values indicate statistically significant *p*-value < 0.05. AAA: abdominal aortic aneurysm; EVAR: endovascular abdominal aortic aneurysm repair; OAR: open aneurysm repair.

**Table 4:** Cox regression analysis for the association between miR-1-3p expression and postoperative mortality of AAA patients through 5-year follow-up.

Variable	Univariate		Multivariate ^a^	
	*p* Value	HR (95% CI)	*p* Value	HR (95% CI)
miR-1-3p expression per unit increase	**0.024**	0.507 (0.281–0.914)	**0.030**	0.496 (0.264–0.933)
miR-1-3p expression				
High levels		1 (Ref)		1 (Ref)
Low levels	**0.002**	3.624 (1.577–8.324)	**0.004**	3.498 (1.478–8.276)

Bold values indicate statistically significant *p*-value < 0.05. ^a^, *p* for association was adjusted by age, renal insufficiency, maximum AAA diameter, presence of surgical complications and presence of postoperative renal dysfunction. AAA: abdominal aortic aneurysm; HR: hazard ratio; CI: confidence interval.

**Table 5:** Effects of rs2155975 and rs4591246 on the expression of serum miR-1-3p in AAA patients.

Variable	rs2155975		*p* Value	rs4591246		*p* Value
	AA	AG + GG		GG	AG + AA	
miR-1-3p expression	1.17 ± 0.67	1.19 ± 0.95	0.905	1.18 ± 0.81	1.18 ± 0.93	0.969
High levels, n (%)	26 (59.1%)	39 (38.6%)	**0.029**	36 (55.4%)	29 (36.3%)	**0.029**
Low levels, n (%)	18 (40.9%)	62 (61.4%)		29 (44.6%)	51 (63.8%)	

Bold values indicate statistically significant *p*-value < 0.05. AAA: abdominal aortic aneurysm.

## Data Availability

The data that support the findings of this study are available from the corresponding author upon reasonable request.

## Supplementary Materials

The following supporting information can be downloaded at: https://www.mdpi.com/article/10.3390/jcm12030946/s1, Table S1: Univariate Cox regression analysis for the prognostic impact of clinical features on all-cause mortality after AAA repair.

## Abbreviations

AAA	abdominal aortic aneurysm
CHB	Chinese Han population
CI	confidence interval
EVAR	endovascular aneurysm repair
HR	hazard ratio
HWE	Hardy–Weinberg equilibrium
MAF	minor allele frequency
miRNA	microRNA
OAR	open aneurysm repair
pri-miRNA	primary miRNA
ROC	receiver operating characteristic
SD	standard deviation
SNP	single nucleotide polymorphism
VSMC	vascular smooth muscle cell
