# Supplementary material for: Association of Genetic Polymorphisms and Serum Levels of miR-1-3p with Postoperative Mortality following Abdominal Aortic Aneurysm Repair
Source: J Clin Med. 2023 Jan 26;12(3):946. doi: 10.3390/jcm12030946 (PMC9917931; doi:10.3390/jcm12030946)
Supplement: Supplementary file 1 [file jcm-12-00946-s001.zip › jcm-2157155-supplementary.pdf]

Table S1. Univariate Cox regression analysis for the prognostic impact of clinical features on all-cause mortality after AAA repair.

Variable	*p* value	HR (95% CI)
Age	＜0.001	1.057 (1.026-1.088)
Male sex	0.497	1.280 (0.628-2.609)
Smoking	0.363	1.275 (0.755-2.151)
Drinking	0.386	0.718 (0.340-1.517)
BMI	0.258	0.953 (0.878-1.036)
Comorbidities		
Coronary artery disease	0.769	1.100 (0.582-2.080)
Cerebrovascular disease	0.128	1.700 (0.859-3.364)
Peripheral artery disease	0.367	1.305 (0.732-2.325)
Renal insufficiency	0.001	2.695 (1.494-4.863)
Atrial fibrillation	0.552	1.361 (0.493-3.762)
Hypertension	0.174	1.505 (0.834-2.713)
Diabetes	0.109	1.692 (0.889-3.220)
Dyslipidemia	0.810	1.076 (0.592-1.955)
Maximum AAA diameter	0.014	1.155 (1.029-1.295)
Presence of surgical complications	0.043	1.933 (1.022-3.656)
Presence of postoperative renal dysfunction	0.083	2.116 (0.908-4.933)
Presence of postoperative respiratory impairment	0.197	1.683 (0.763-3.712)
Presence of reoperation	0.778	1.114 (0.527-2.352)
Surgical type	0.310	0.679 (0.321-1.434)

BMI: body mass index; AAA: abdominal aortic aneurysm; HR: hazard ratio; CI: confidence interval.
